# Minority report: small-scale metagenomic analysis of the non-bacterial kitchen sponge microbiota

**DOI:** 10.1007/s00203-022-02969-9

**Published:** 2022-06-04

**Authors:** Lena Brandau, Susanne Jacksch, Severin Weis, Sylvia Schnell, Markus Egert

**Affiliations:** 1 Faculty of Medical and Life Sciences, Institute of Precision Medicine, Microbiology and Hygiene Group, Furtwangen University, Villingen-Schwenningen, Germany; 2 Research Centre for BioSystems, Land Use, and Nutrition (IFZ), Institute of Applied Microbiology, Justus-Liebig-University Giessen, Giessen, Germany

**Keywords:** Built environment, Kitchen sponge, Microbiome, Virus, *Archaea*, *Eukaryota*, Metagenomic shot-gun sequencing

## Abstract

Kitchen sponges are particularly well known to harbor a high number and diversity of bacteria, including pathogens. Viruses, archaea, and eukaryotes in kitchen sponges, however, have not been examined in detail so far. To increase knowledge on the non-bacterial kitchen sponge microbiota and its potential hygienic relevance, we investigated five used kitchen sponges by means of metagenomic shot-gun sequencing. Viral particles were sought to be enriched by a filter step during DNA extraction from the sponges. Data analysis revealed that ~ 2% of the sequences could be assigned to non-bacterial taxa. Each sponge harbored different virus (phage) species, while the present archaea were predominantly affiliated with halophilic taxa. Among the eukaryotic taxa, besides harmless algae, or amoebas, mainly DNA from food-left-overs was found. The presented work offers new insights into the complex microbiota of used kitchen sponges and contributes to a better understanding of their hygienic relevance.

**Supplementary Information:**

The online version contains supplementary material available at 10.1007/s00203-022-02969-9.

## Introduction

Thousands of microbial species are living in the indoor biome (Martin et al. 2015). They are brought inside through humans, food, clothes, and pets, and are also spread by insects, through water or air (Gilbert and Stephens 2018; Kelley and Gilbert 2013; Prussin et al. 2015). Using a wide variety of cleaning measures and (antimicrobial) cleaning agents, extreme environmental conditions are created, forming special niches for microbial life leading to unique microbial associations in the different sites of a modern household (Gilbert and Stephens 2018; Kelley and Gilbert 2013). Used kitchen sponges probably represent inanimate objects with the highest bacterial loads in households with average counts of ~ 10^7^–10^10^ CFUs per sponge (Cardinale et al. 2017; Marshall et al. 2012; Rossi et al. 2012). As the sponges are highly porous, usually wet, and rich in nutrients, they form a perfect breeding ground for bacteria and other microorganisms (Donofrio et al. 2012). Cardinale et al. investigated the bacterial community composition of used kitchen sponges and identified *Proteobacteria*, *Bacteroidetes*, and *Actinobacteria* as the dominant phyla (Cardinale et al. 2017), which corroborated studies on the overall kitchen microbiota (Flores et al. 2013). They also discovered species of the genera *Acinetobacter*, *Chryseobacterium*, and *Moraxella*, which were categorized as organisms of risk group 2, i.e., as potentially pathogenic (Cardinale et al. 2017). Other studies reported potentially pathogenic bacteria in kitchen sponges, too, such as *Klebsiella* spp. (Adiga et al. 2012; Marshall et al. 2012; Osaili et al. 2020), *Escherichia coli* (Adiga et al. 2012; Marshall et al. 2012), or *Salmonella* spp. (Rossi et al. 2012). Kitchen sponges are not only of hygienic relevance because they produce bad smell, but also because they can transfer pathogens to kitchen surfaces through cross-contamination (Rossi et al. 2013), potentially leading to foodborne illnesses (Greig and Ravel 2009). So far, kitchen sponge studies largely focused on the bacterial community composition, and only a few of them briefly mentioned the presence of other microbial taxa, such as yeasts and molds (Jacksch et al. 2020; Marotta et al. 2018), archaea (Flores et al. 2013; Jacksch et al. 2020), and eukaryotes or viruses (Jacksch et al. 2020). 16S or 18S rRNA gene amplicon sequencing is an easy way to detect microorganisms and allows great insights into their community composition, including yet uncultured taxa, making it the most common method to analyze microbial communities (Gilbert and Stephens 2018; Kelley and Gilbert 2013). In metagenomic shot-gun sequencing (MGS), overlapping regions of genes are sequenced, enabling the simultaneous detection of various organisms and their genetic content without PCR-amplification bias. However, this approach is more expensive, and data analysis is more complex (Ranjan et al. 2016), partly explaining, why the non-bacterial kitchen sponge microbiota might have been neglected so far.

In this study, we used MGS to obtain a more complete picture of the community composition and potential hygienic relevance of the kitchen sponge microbiota. Since bacteriophages and other viral particles are comparatively small, a filter step for virus enrichment was integrated in the procedure of nucleic acid extraction. Our results provide the initial insights into the presence of viruses, archaea, and eukaryotes in used kitchen sponges, and offer a basis for an evaluation of their hygienic relevance.

## Materials and methods

### Sample collection, preparation, and shot-gun sequencing

Used kitchen sponges (*n* = 5) were obtained from regular households of 1–2 people where the sponges where mainly used for washing dishes over a period of 3–10 weeks (Table 1). Sponge A came from a student dormitory with 12 students using the sponge. Detailed information about the usage of each kitchen sponge can be found in the supplementary material (Tab. S1).

**Table 1:** Key data of the analyzed sponges

Sponge	Application	Frequency of use	Time period
A	Washing dishes	> 7–9×/week	3–5 weeks
B	Washing dishes	Daily, 2–3×	2.5 months
C	Washing dishes	Daily, 1–2×	3 weeks
D	Mainly kitchen surfaces	Daily, 1×	1–2 months
E	Washing dishes	Daily, 2–3×	3.5 weeks

For each sponge, four cross sections of 0.5 cm width were each vortexed in 10 ml of sterile phosphate-buffered saline (PBS), pH 7.4 (Chazotte 2012), for 20 min to bring the microbial material into solution. Liquids were then collected from each cross section by squeezing them with a sterile syringe. Per section, 3 ml of each suspension were used as “unfiltered” samples, and the rest was filtered with a 0.45 µm pore size-calcium acetate-syringe filter to remove microbial cells and increase the concentration of smaller, viral particles. A negative control (NC) was prepared without sponge tissue and split into an unfiltered and filtered subsample, too.

DNA was extracted from the samples using the Monarch Genomic DNA Purification Kit (New England Biolabs, Frankfurt am Main, Germany) according to the manufacturer’s instructions for purification from saliva (Taron et al. 2020). The DNA was eluted twice from the column membranes using 50 µl elution buffer, incubation at 60 °C for 10 min, centrifugation, and reapplying the same 50 µl to the membrane. DNA was quantified with a NanoPhotometer P360 (Implen, München, Germany) and a Qubit 2.0 Fluorometer (Life Technologies, Carlsbad, CA, USA) using the Qubit dsDNA HS Assay Kit (Thermo Fisher Scientific, Waltham, MA, USA).

The two highest concentrated sample pairs (unfiltered and filtered) per sponge were selected for MGS. Library preparation was performed with the Illumina DNA Prep kit (Illumina, San Diego, CA, USA) according to the manufacturer’s instructions using 20 ng of genomic DNA per sample as input and the IDT for Illumina UD Indexes Plate A/Set 1 (Illumina) for the sample-wise indexing. Quality and size profile of the finished libraries were checked using a 2100 Bioanalyzer Instrument with the Agilent DNA 1000 kit (both from Agilent Technologies, Santa Clara, CA, USA) according to the manufacturer’s instructions. Quantification of the libraries was performed with the Qubit dsDNA HS Assay Kit as described above.

Libraries were pooled, diluted to a final concentration of 10 pM with 5% PhiX, and sequenced on an Illumina MiSeq platform using the 500 cycles MiSeq v2 Reagent Kit (Illumina) with a 2 × 250 bp sequencing program.

### Data preparation and statistical analysis

Quality control (QC) of the reads was performed with FastQC (Andrews 2020) and MultiQC (Ewels et al. 2016). Quality trimming was performed using Sickle (Joshi and Fass 2011) with a Quality (Q)-Score cut-off at Q30 and a minimum length of 50 bp for sequences to keep. Human and rRNA reads were removed based on the genome sequence of the Genome Reference Consortium Human build 38 patch release 13 (GRCh38.p13) (GENECODE 2020) and the rRNA database of SortMeRNA (Kopylova et al. 2012), respectively, using Bowtie2 (Langmead and Salzberg 2012). De-novo assembly was performed with ABySS (Simpson et al. 2009) using *k*-mers from 32 to 96 in steps of eight. The assembly was additionally performed with MEGAHIT (Li et al. 2015) and assembly statistics were computed using QUAST (Gurevich et al 2013). To figure out the best alignment produced by the different ABySS *k*-mers, the assemblies, fitting best to the ones of MEGAHIT, were aligned to the human- and rRNA-reduced reads using Bowtie2. With BLAST + (Camacho et al. 2009), the best matching assembly was searched for the present taxonomy using the nucleotide BLAST “blastn” with a megablast approach and the nucleotide (nt) database. The e-value was set to 1e-5 and to not only receive the first best hit the parameter “max_target_seqs” was set to 5. The statistics software R v.4.0.3 (R Core Team 2020) and RStudio v.1.4.1103 (RStudio Team 2020) were used for further processing of the data. Taxonomy was assigned with the package taxonomizr v.0.6.0 (Sherrill-Mix 2021) based on the accession numbers of the National Center for Biotechnology Information (NCBI). With a parallel approach, using the packages doParallel v.1.0.16 (Microsoft Corporation and Weston 2020a, b), foreach v.1.5.1 (Microsoft Corporation and Weston 2020b), and doMC v.1.3.7 (Revolution Analytics and Weston 2020), the BLAST results were filtered for the best hit. Contigs with no alignment hit were removed.

Statistical analysis and graphical visualization were performed in R using mainly the packages phyloseq v.1.32.0 (McMurdie and Holmes 2013), ggplot2 v.3.3.3 (Wickham 2016), and ggpubr v.0.4.00 (Kassambara 2020). Based on the three present domains and the virus group, the data set was divided into four smaller data sets, and contigs, not assigned to at least one individual sample, were removed. Taxa of the same type were agglomerated on the taxonomic levels. The dataset was reduced to the top ten taxa to present the abundance of contigs in a plot. Alpha diversity was calculated for the filter types (unfiltered, filtered) and individual sponges using the common alpha-diversity measures of the observed unique taxa, Shannon index, and Simpson index. One-way analysis of variance (ANOVA) was used to compare the results, followed by p-value adjustment according to Holm (Holm 1979). Multiple pairwise-comparison was conducted as post hoc test using Tukey’s Honestly Significant Difference (HSD) (Yandell 1997) with a 95% family-wise confidence level and already adjusted p values.

## Results

### Sequencing data

On average, sequencing resulted in 549,764 raw sequences per sponge sample from which 473,971 remained after quality trimming. The filtered samples delivered 1.7% more reads than the unfiltered samples. The average amount of human reads that were removed was 0.37% and for rRNA reads 0.75%. The MEGAHIT assembly revealed an N50 contig length of 1232 bp. Similar values were achieved with *k*-mer 56, which reached the highest alignment rates in the assembly QC and was used for further analysis.

### Taxonomic composition of the kitchen sponges

42.9% of the assembly contigs were assigned to a taxonomy by BLAST. After removal of contigs not assigned to at least one sample, 782,192 contigs remained in total. On average, around 98% of the sequences per sponge were assigned to the domain *Bacteria*, ~ 1.6% to *Eukaryota*, ~ 0.14% to viruses and ~ 0.007% to *Archaea* (absolute number of sequences affiliated with the respective domain, divided by all assigned sequences, averaged over the five investigated sponges). The bacterial community composition was dominated by the classes *Gammaproteobacteria*, *Flavobacteriia*, and *Alphaproteobacteria*. The NCs only contained bacterial taxa of the species *Cutibacterium acnes* (phylum *Actinobacteria*), and *Sphingobium amiense* and *Cellvibrio japonicus* (phylum *Proteobacteria*), which probably originated from the used reagents and kits (Salter et al. 2014). Notably, other domains or viruses were not detected in the NCs. In the following, for the sake of simplicity, the taxonomic ranks are generally referred to as phyla, class, order, family, and genus, even though viruses and eukaryotes may be classified into further subgroups (Burki et al. 2020; International Committee on Taxonomy of Viruses 2020).

**Fig. 1:**
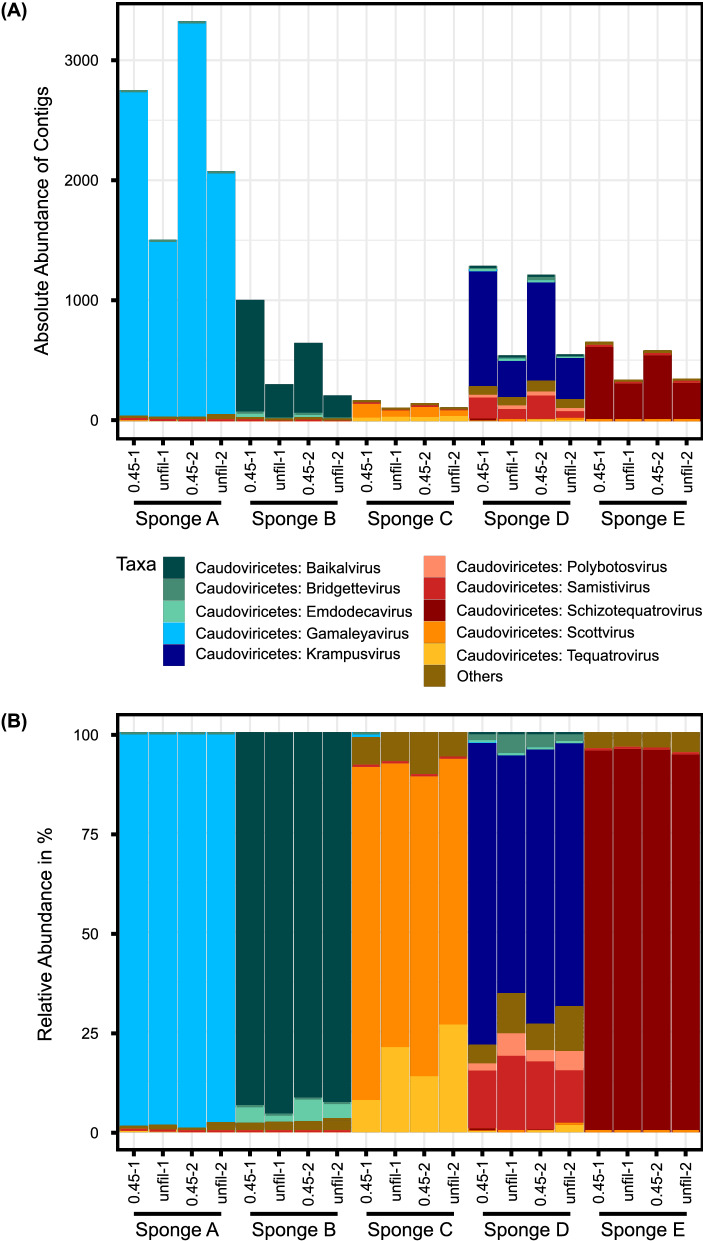
Taxonomic composition of viruses. The figure shows the top ten most abundant genera with corresponding classes of the assigned viral taxa, present in the unfiltered (“unfil”) and filtered (“0.45”) kitchen sponge samples, as delivered by Illumina-based metagenomic shot-gun sequencing. The abundance is shown as **A** the absolute abundance of contigs and **B** the relative abundance in %. The remaining taxa were grouped as “Others”. In the sponge negative controls, no viral taxa were present. The graph was generated with agglomerated data created with the function glom_taxa of the R package phyloseq

To viruses, 169 unique contigs were assigned, representing 4 phyla, 5 classes, 7 orders, 10 families, and 56 genera. The most common class, with a relative abundance of 94% over all sponges, was *Caudoviricetes* with the order *Caudovirales* (Fig. 1) and the families *Podoviridae*, *Myoviridae*, *Autographiviridae,* and *Siphoviridae*, whereby each sponge was dominated by a different virus family. The same effect showed up for the genus level, whereby a different dominantly present virus genus was present with specific species for each sponge: *Gamaleyavirus* with *Escherichia virus EC1-UPM* and *Escherichia virus APEC7*, *Baikalvirus* with *Pseudomonas virus PaBG*, *Scottvirus* with *Sphingomonas virus Scott*, *Krampusvirus* with different *Microbacterium phages,* and *Schizotequatrovirus* with *Vibrio virus KVP40*. The bacterial hosts of these viruses were also found in the respective sponge samples. Comparing the unfiltered and filtered samples, the latter one showed distinctly higher absolute abundances of present contigs.

**Fig. 2:**
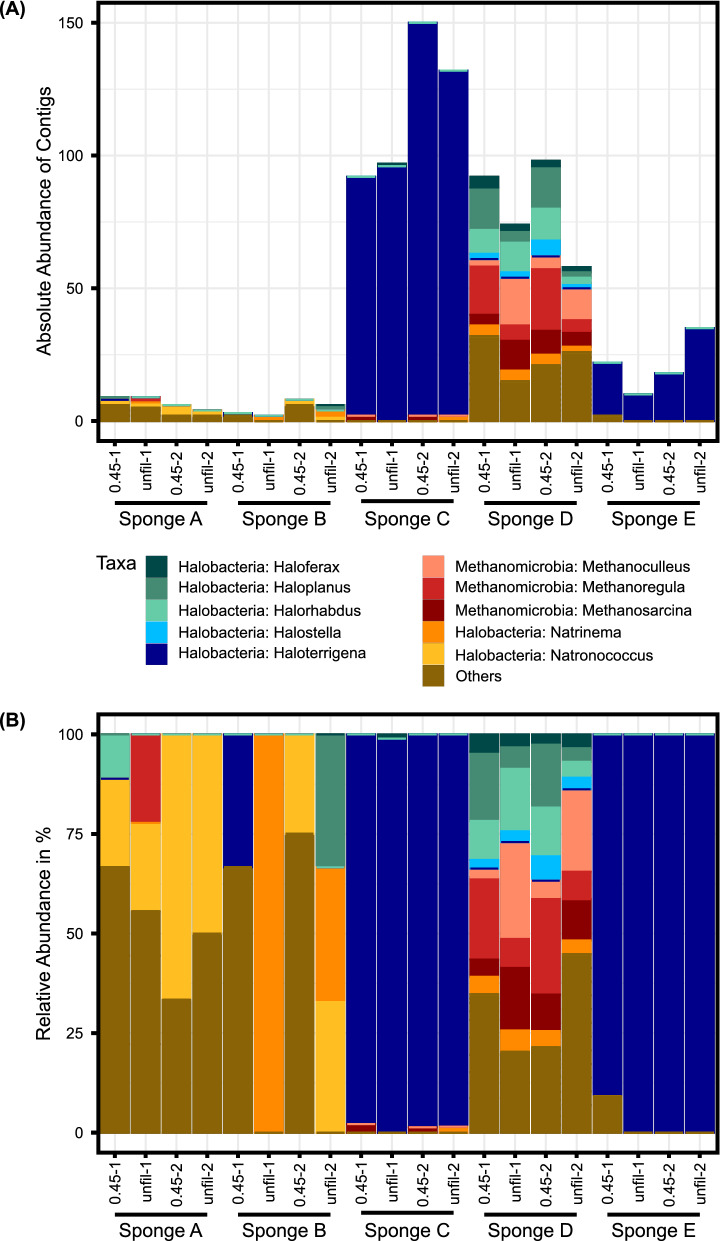
Taxonomic composition of *Archaea*. The figure shows the top ten most abundant genera with corresponding classes of the assigned archaeal taxa, present in the unfiltered (“unfil”) and filtered (“0.45”) kitchen sponge samples, as delivered by Illumina-based metagenomic shot-gun sequencing. The abundance is shown as **A** the absolute abundance of contigs and **B** the relative abundance in %. The remaining taxa were grouped as “Others”. In the sponge negative controls, no archaeal taxa were present. The graph was generated with agglomerated data created with the function glom_taxa of the R package phyloseq

To the domain *Archaea*, 63 unique contigs were assigned, representing 4 phyla, 9 classes, 14 orders, 19 families, and 38 genera. The most common class, present in all five sponges, was *Halobacteria* with a relative abundance of around 57% overall (Fig. 2). In sponges C and E, only two-to-four different *Archaea* genera were found, dominated by the genus *Haloterrigena* from the order *Natrialbales*. The present *Haloterrigena* species were *Haloterrigena* sp. YPL8 and an uncultured *Haloterrigena* species. In sponge A, the species *Natronococcus occultus* made up one-third of the identified taxa. The same species was present in sponge B with a relative abundance of 21%, as well as the genus *Halorubrum* with the species *H.* sp. PV6 and *H. lacusprofundi*, and the genus *Natrinema* with various species. Beside *Halobacteria*, another common class in sponge D was *Methanomicrobia*. Overall, this sponge showed a more diverse composition of *Archaea* genera than the other sponges. Similar to the viruses, the filtered samples here showed slightly higher absolute abundances of contigs than the unfiltered samples.

**Fig. 3:**
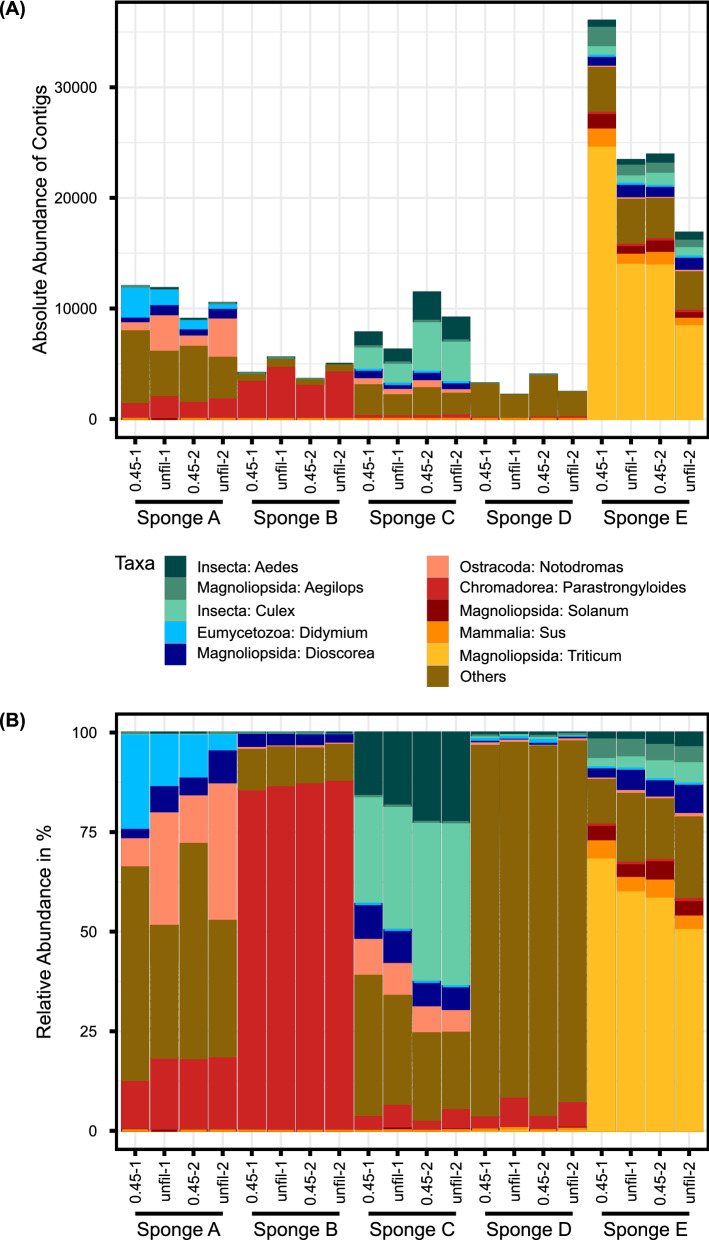
Taxonomic composition of *Eukaryota*. The figure shows the top ten most abundant genera with corresponding classes of the assigned eukaryotic taxa, present in the unfiltered (“unfil”) and filtered (“0.45”) kitchen sponge samples, as delivered by Illumina-based metagenomic shot-gun sequencing. The abundance is shown as **A** the absolute abundance of contigs and **B** the relative abundance in %. The remaining taxa were grouped as “Others”. In the sponge negative controls, no eukaryotic taxa were present. The graph was generated with agglomerated data created with the function glom_taxa of the R package phyloseq

For the domain *Eukaryota*, 32,137 unique contigs were identified, representing 29 phyla, 98 classes, 311 orders, 584 families, and 902 genera. Most common, with a relative abundance of 73% overall, was the class *Magnoliopsida* (Fig. 3), dominantly present in sponge E with the genus *Triticum*. Other non-microbial genera were, for example, *Culex* and *Aedes* of the class *Insecta*, or *Notodromas* of the class *Ostracoda*. In sponge B, most taxa were assigned to the genus *Parastrongyloides* with the species *P. trichosuri* from the class *Chromadorea*. However, this class only accounted for around 5% of the present *Eukaryota*-classes of all sponges. Other identified genera represented molds like *Didymium*, *Fusarium* or *Cavenderia*, algae like *Trebouxia* or *Plocamiocolax*, or ciliates like *Tetrahymena*. For most of the samples, the filtered ones showed again slightly higher abundances of contigs than the unfiltered ones.

A detailed overview of the abundance of the top ten taxa and their occurrence in the single kitchen sponges can be found in the supplementary material (Tab. S1).

For all domains (*Bacteria*, *Archaea*, *Eukaryota*) and the viruses, the calculated alpha-diversity measures (observed unique taxa, Shannon- and Simpson index) showed no statistical significance (ANOVA, *p* > 0.05) for the difference between the filtered and unfiltered samples. However, the difference in alpha-diversity between the five kitchen sponges revealed statistical significance (ANOVA, *p* < 0.001) in all calculated alpha-diversity measures. Detailed results of the alpha-diversity statistics can be found in the supplementary material (Fig. S1). Sponge D showed the highest diversity with respect to viruses and *Archaea,* which was confirmed by Tukey’s HSD, revealing statistical significance (*p* < 0.001) for the difference in alpha-diversity of sponge D compared to the other sponges. For the viruses, the sponges B and C also showed higher alpha-diversities. The alpha-diversity of eukaryotes was highest in sponge E. Tukey’s HSD indicated statistical significance (*p* < 0.001) for the difference in alpha-diversity between sponge E and the other sponges for almost all alpha-diversity measures. Furthermore, the difference between sponge B and C revealed to be statistically significant (*p* < 0.001) by Shannon and Simpson index.

## Discussion

The spread of microorganisms in households and especially in kitchens has been studied increasingly in recent years (Donofrio et al. 2012; Flores et al. 2013; Prussin et al. 2015; Rintala et al. 2008; Speirs et al. 1995). Kitchen sponges were examined as well-known microbial hot spots in the built and kitchen environment, too, albeit with a clear focus on bacteria (Cardinale et al. 2017; Jovanovska et al. 2018; Marotta et al. 2018; Møretrø et al. 2021; Osaili et al. 2020). However, non-bacterial microorganisms such as viruses, archaea, and eukaryotes might also be of hygienic importance (Jacksch et al. 2020). Our study provides an initial impression of this non-bacterial microbiota in used kitchen sponges.

The statistically significant differences between the alpha-diversities of the five analyzed kitchen sponges suggest that the household environment could also affect the presence of viruses, archaea, and eukaryotes in addition to bacteria (Cardinale et al. 2017), forming an individual community for every sponge. Our data suggest that the non-bacterial microbiota only makes up a small fraction of the total sponge microbiota (~ 2%, based on assigned sequences), and that it is mainly composed of non-pathogenic bacteriophages, and halophilic and methanogenic archaea, while the eukaryotic DNA mainly represented non-microbial organisms, including DNA from food residues. Clearly, our results should not be overinterpreted, as only five kitchen sponges were examined here. In addition, our approach was DNA-based, not allowing any differentiation between living and dead organisms, and also discriminating RNA-viruses. Finally, the used DNA extraction and metagenomics analysis kit is, based on information of the manufacturer, not validated for the use with viruses. Therefore, we cannot exclude that most of the detected virus (phage) sequences were prophages inserted in the bacterial genomes and that free DNA viruses might have been discriminated due to insufficient lysis. Nevertheless, we believe that our data provide valuable first insights into the non-bacterial kitchen sponge microbiota.

Overall, the dominant virus order of the kitchen sponges was *Caudovirales* of the class *Caudoviricetes*. These belong to the so-called tailed phages that represent the largest of all known virus groups (Ackermann 1996; Ackermann and Prangishvili 2012) and was also the dominant virus group in the sponge study of Jacksch et al. (Jacksch et al. 2020). *Caudovirales* mainly infect *Enterobacteria* but generally appear in a wide variety of bacteria (Maniloff and Ackermann 1998). Interestingly, some species of the families *Myoviridae* and *Siphoviridae* also infect halophilic or methanogenic archaea (Ackermann 1998; Maniloff and Ackermann 1998; Witte et al. 1997), from which *Methanobrevibacter* was also present in one of the investigated sponges. The identified *Escherichia virus EC1-UPM* is known to infect *Escherichia coli* O78:K80 which causes colibacillosis in poultry (Gan et al. 2013). We did not analyze the specific *E. coli* strains in our samples, but the virus might be in the kitchen sponge due to contaminated poultry products. *Pseudomonas virus PaBG* was also detected in the investigated kitchen sponges. It specifically infects *Pseudomonas aeruginosa*, which is often known as antibiotic-resistant pathogen causing nosocomial infections, but also occurs in many other wet habitats (Evseev et al. 2020; Robert Koch-Institut 2017). *Pseudomonas aeruginosa* was indeed present in the sponge samples. *Vibrio virus KVP40* is known to infect *Vibrio cholerae*, *V. parahaemolyticus,* and other species of the family *Vibrionaceae* (Matsuzaki et al. 1992), which was also present in the kitchen sponges. However, the *Vibrio cholerae* species were most likely not the infectious ones (Schwartz et al. 2019). As kitchen sponges host many bacteria (Cardinale et al. 2017), it was expectable to detect bacteriophages here. These phages might influence bacterial community composition and diversity through selective lysis or horizontal gene transfer (Fuhrman 1999). Especially, the latter might play a role in the transfer of antibiotic resistance genes (Balcazar 2014; Lerminiaux and Cameron 2019).

The identified *Archaea* species predominantly belonged to the class *Halobacteria*, which is a halophilic taxon and one of the largest groups within its domain (Gupta et al. 2015). Halobacteria usually prefer environments with salt concentrations of 20% and form biofilms (Pfeifer 2017), which enables them to withstand such extreme conditions. The presence of these halophils inside the kitchen sponges suggests a potential retention of salt in the sponges maybe resulting from a salty human diet (World Health Organization 2021). Cardinale et al. already detected bacterial biofilm formation in kitchen sponges (Cardinale et al. 2017), clearly marking sponges as a favorable site for this protective measure against environmental stressors. In our study, the analyzed kitchen sponges contained halophiles of the genus *Natrinema*, from which some live strictly aerobic and others anaerobic (McGenity et al. 1998; Xin et al. 2000), and the species *Halorubrum lacusprofundi*, which is additionally psychrotolerant (Anderson et al. 2016). Furthermore, the haloalkaliphilic archaeon *Natronococcus occultus* was detected, that prefers a pH range of 8.5–11 in addition to the extreme salt conditions (Tindall et al. 1984). Corroborating the results of Jacksch et al., we also identified methanogens (Jacksch et al. 2020) like the *Archaea*-class *Methanomicrobia*. Their intolerance to oxygen (Laskar et al. 2018) points to anaerobic conditions inside the sponges, likely caused by waterlogging, biofilm formation, and the oxygen consumption by the large number of bacteria. Members of the class *Methanomicrobia* are typically found in marine or freshwater sediments, and several have adapted to a variety of conditions from low to high pH, different temperatures, nutrients, or salt contents (Browne et al. 2017; Dworkin and Falkow 2006). Interestingly, humans also represent a possible source of *Halobacteria* and *Methanobacteria*, as they have been detected on human skin, too (Umbach et al. 2021).

For many eukaryotes, interpretation of the presence of their DNA was more difficult. Most of the assigned sequences probably stem from food left-overs or DNA residues on tropical fruits or similar objects, but not from living organisms. Examples are the parasitic roundworm *Parastrongyloides trichosuri* that infects the brushtail possum, living in Australia and New Zealand (Garcia 1999; Hunt et al. 2016), or species of the mosquito genera *Culex* and *Aedes*, that are not occurring in southern Germany (Biogents 2016; Samy et al. 2016). We additionally identified the genera *Didymium* and *Cavenderia* of the class *Eumycetozoa*, that comprises groups like true slime molds, or protosteloid amoebae (Stephenson and Schnittler 2017). They can be found in terrestrial ecosystems or freshwater and function as bacterivores, predators of fungi and bacteria, and recycler of nutrients, making a domestic kitchen sponge an attractive habitat for them (Spiegel et al. 2017). The algal families *Trebouxiophyceae* and *Florideophyceae*, as well as the ciliate *Tetrahymena* occur in different waters and terrestrial environments (Leliaert et al. 2012; Ruehle et al. 2016; Yoon et al. 2017) and might have entered the sponges via tap water. The detected fungal genus *Fusarium* comprises mycotoxin producer affecting mainly wheat and maize but also other crops (Munkvold 2017) and might stem from left-overs of grain products.

Cleaning and dietary habits might have led to the observed differences in taxonomic diversity between the five kitchen sponges (Cardinale et al. 2017), pointing to a “unique microbial fingerprint” (Lax et al. 2014). However, a definitive statement requires a larger number of samples. With regard to the filter step used, especially for the viral taxonomy, the filtered samples showed a higher absolute abundance of assigned contigs than the unfiltered samples. This might be due to an enrichment of viral particles and free viral DNA with a simultaneous reduction in bacterial cells through the filter step (Hall et al. 2014). Such filtration is used as a preparative step in virus enrichment methods via ultracentrifugation (Lawrence and Steward 2010; Thurber et al. 2009). The slight enrichment of archaeal or eukaryotic DNA might result from free DNA of damaged cells.

In summary, our small-scale study suggests that kitchen sponges harbor a small, but diverse and biologically very interesting non-bacterial microbiota comprising bacteriophages, archaea, fungi, algae, and other small eukaryotes, which probably interact with the bacterial microbiota. However, from a hygienic point of view, e.g., regarding the spread of infectious diseases, this microbiota is probably of minor importance. Follow-up experiments with more sponges will enable deeper statistical analyses and hypothesis testing, e.g., regarding the influence of cleaning measures and other environmental factors on the total sponge microbiota. In addition, RNA-based analysis will help to unravel, the active (living) fractions of the microbiota. Virome analyses should target RNA-viruses in addition to DNA viruses and should include additional enrichment steps, e.g., using density gradient ultracentrifugation.

## Supplementary Information

Below is the link to the electronic supplementary material.

Supplementary file1 Fig S1: Comparison of the alpha-diversity between the different kitchen sponges. (DOCX 244 KB)

Supplementary file2 Table S1: Sample metadata and abundance of top 10 viral, archaeal, and eukaryotic taxa. (XLSX 20 KB)

## Data Availability

Sequences generated and analyzed here are accessible at the European Nucleotide Archive (ENA) under the accession number PRJEB49259. Subject metadata is included in the supplementary Table S1. Only open-source code from the cited R (v.4.0.3) packages (taxonomizr v.0.6.0, doParallel v.1.0.16, foreach v.1.5.1, doMC v.1.3.7, phyloseq v.1.32.0, ggplot2 v.3.3.3, ggpubr v.0.4.0) was used, using either the default settings or the settings stated in the methods section. Further information for clarification is available from the corresponding author on reasonable request.
