# Supplementary material for: Minority report: small-scale metagenomic analysis of the non-bacterial kitchen sponge microbiota
Source: Arch Microbiol. 2022 Jun 4;204(7):363. doi: 10.1007/s00203-022-02969-9 (PMC9167186; doi:10.1007/s00203-022-02969-9)
Supplement: Supplementary file 1 — Supplementary file1 Fig S1: Comparison of the alpha-diversity between the different kitchen sponges. (DOCX 244 KB) [file 203_2022_2969_MOESM1_ESM.docx]

**Minority report - Small-scale metagenomic analysis of the non-bacterial kitchen sponge microbiota**

**Archives of Microbiology**

**Lena Brandau^1^ [ORCiD: 0000-0003-3102-7241], Severin Weis^1^, Susanne Jacksch^1^, Sylvia Schnell^2^,Markus Egert^1,*^ [ORCiD: 0000-0003-2402-3322]**

^1^ Faculty of Medical and Life Sciences, Institute of Precision Medicine, Microbiology and Hygiene Group, Furtwangen University, Villingen-Schwenningen, Germany

^2^ Research Centre for BioSystems, Land Use, and Nutrition (IFZ), Institute of Applied Microbiology, Justus-Liebig-University Giessen, Giessen, Germany

^*^ Correspondence: [Markus.Egert@hs-furtwangen.de](mailto:Markus.Egert@hs-furtwangen.de)


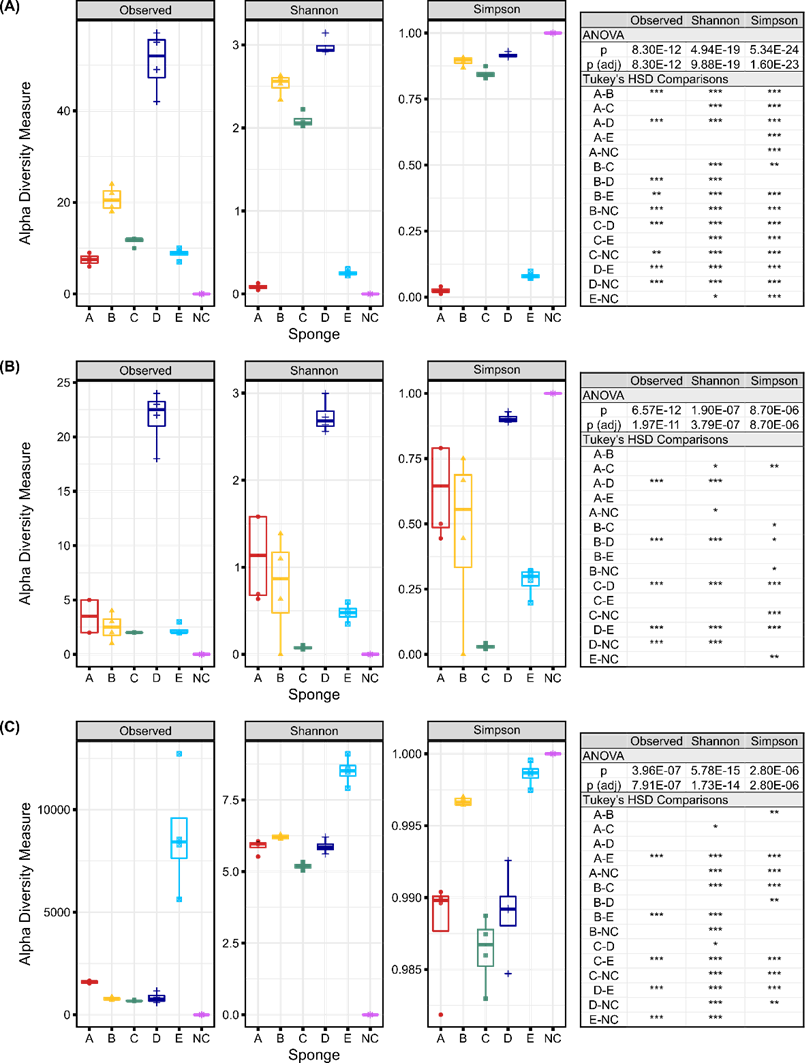


**Fig S1** Comparison of the alpha-diversity between the different kitchen sponges. Alpha-diversity was calculated for the (A) viral, (B) archaeal, and (C) eukaryotic taxa of all samples per kitchen sponge (“A-E”) and the negative control (“NC”). Statistical analysis was done by one-way ANOVA with follow-up Holm p-value adjustment, indicating significant results for all used diversity measures (Observed unique taxa, Shannon-, and Simpson-index). As post-hoc test, Tukey’s HSD was used of which the adjusted p-values are shown in the table as asterisks: p < 0.05 = *, p < 0.01 = **, p < 0.001 = ***. Contigs, not assigned to at least one sample, were removed. The NC contained no viral, archaeal, or eukaryotic taxa.
